# Nosocomial Transmission of *Plasmodium falciparum* Malaria, Spain, 2024 (Response)

**DOI:** 10.3201/eid3109.251210

**Published:** 2025-09

**Authors:** Manuel F. Liroa Romero, Maite Ruiz Pérez de Pipaón, Maria D. Navarro Amuedo, Jose M. Rubio Muñoz, Jose M. Jiménez-Hoyuela, Jose M. Cisneros

**Affiliations:** University of Seville, Seville, Spain (M.F. Liroa Romero, M. Ruiz Pérez de Pipaón, M.D. Navarro Amuedo, J.M. Jiménez-Hoyuela, J.M. Cisneros); Hospital Universitario Virgen del Rocío, Seville (M.F. Liroa Romero, M. Ruiz Pérez de Pipaón, M.D. Navarro Amuedo, J.M. Jiménez-Hoyuela, J.M. Cisneros); CIBERINFEC Instituto de Salud Carlos III, Madrid, Spain (J.M. Rubio Muñoz, J.M. Cisneros)

**Keywords:** malaria, Plasmodium falciparum, vector-borne infections, acquired malaria, parenteral transmission, parasites, Spain

**In Response:** We appreciate the opportunity to respond to the letter from Dr. Perales (*1*) regarding our article on a case of nosocomial transmission of *Plasmodium falciparum* during a thyroid scintigraphy (*2*). The author of the letter proposes as an alternative mechanism the nosocomial transmission of the parasite by contact with blood on gloves not changed between patients. We respectfully disagree because the mechanism he proposes lacks scientific basis; no evidence of malaria transmission by such contact has been documented. On the contrary, nosocomial transmission of *P. falciparum* by parenteral route is well demonstrated, through blood transfusions, transplants (*3*), or invasive medical procedures involving exposure to contaminated blood (*3*), as occurred in our case (*2*).
